# Effect of ZnO Nanoparticle Content on the Structural and Ionic Transport Parameters of Polyvinyl Alcohol Based Proton-Conducting Polymer Electrolyte Membranes

**DOI:** 10.3390/membranes11030163

**Published:** 2021-02-26

**Authors:** Omed Gh. Abdullah, Yahya A. K. Salman, Dana A. Tahir, Gelas M. Jamal, Hawzhin T. Ahmed, Azhin H. Mohamad, Auday K. Azawy

**Affiliations:** 1 Advanced Materials Research Laboratory, Department of Physics, College of Science, University of Sulaimani, Kurdistan Region 46001, Iraq; gelas.jamal@univsul.edu.iq; 2 Department of Physics, College of Science, University of Mosul, Mosul 41002, Iraq; kareem200138@yahoo.com; 3 Department of Physics, College of Science, University of Halabja, Kurdistan Region 46006, Iraq; dana.tahir@uoh.edu.iq (D.A.T.); auday@mail.ru (A.K.A.); 4 Charmo Center for Research, Training and Consultancy, Charmo University, Kurdistan Region 46023, Iraq; hawzhin.taha@charmouniversity.org; 5 Department of Physics, College of Education, University of Sulaimani, Kurdistan Region 46001, Iraq; azhin.mohamad@univsul.edu.iq

**Keywords:** ZnO-NPs, proton-conducting, impedance, conductivity, transport parameters

## Abstract

Proton conducting nanocomposite solid polymer electrolytes (NSPEs) based on polyvinyl alcohol/ammonium nitrate (PVA/NH_4_NO_3_) and different contents of zinc oxide nanoparticles (ZnO-NPs) have been prepared using the casting solution method. The XRD analysis revealed that the sample with 2 wt.% ZnO-NPs has a high amorphous content. The ionic conductivity analysis for the prepared membranes has been carried out over a wide range of frequencies at varying temperatures. Impedance analysis shows that sample with 2 wt.% ZnO-NPs has a smaller bulk resistance compared to that of undoped polymer electrolyte. A small amount of ZnO-NPs was found to enhance the proton-conduction significantly; the highest obtainable room-temperature ionic conductivity was 4.71 × 10^−4^ S/cm. The effect of ZnO-NP content on the transport parameters of the prepared proton-conducting NSPEs was investigated using the Rice–Roth model; the results reveal that the increase in ionic conductivity is due to an increment in the number of proton ions and their mobility.

## 1. Introduction

Research on proton-conducting solid polymer electrolytes (SPEs) over the past few decades has aimed to provide high-performance and stable electrochemical devices, such as electrochemical double-layer capacitors, light-emitting electrochemical cells, solid-state batteries, and fuel cells [1,2,3]. The proton transport in SPEs can be designated based on three mechanisms: hopping, diffusion, and transport associated with polymer chain segmental movement [4]. The ion hopping mechanism and ion transport by segmental motions are more favored at higher temperatures [5]. To investigate the proton conduction mechanism of a system, the ionic conductivity is typically characterized in terms of temperature [6].

Nonetheless, the greatest drawback of proton-conducting SPEs is their low ionic conductivity at room temperature, which restricts their practical applications in energy storage devices [7]. In recent years, significant efforts have been dedicated to enhancing ionic conduction in proton-conducting SPEs by different approaches, including polymer blending, copolymerization, the addition of plasticizers, and the incorporation of nano-sized inorganic fillers to the system such as carbon nanotubes, reduced graphene oxide, and metal oxide. Among these approaches, the dispersion of a small amount of inorganic nano-sized fillers into the polymer electrolyte matrix has captured escalating interest by many researchers due to their high efficiency in improving the room-temperature ionic conductivity of an electrolyte system [8,9]. The overall impact of presenting nano-sized inorganic fillers in SPEs depends on several factors, such as the type, shape, size, and concentration of nano-filler, as well the way in which the fillers are distributed and dispersed in the matrix [10,11].

Polyvinyl alcohol (PVA) has been extensively used as the host for electrolyte systems due to its hydrophilic properties, non-toxicity, water-solubility, biocompatibility, biodegradability, low cost, and good film-forming properties [12,13]. The hydroxyl (O–H) groups of PVA help in the dissociation of salts at high concentrations to form an ionic medium as a result of hydrogen bonding, which makes the PVA matrix a potential candidate in electronics and optoelectronics applications [14,15].

Due to the polar nature of PVA, the ions of dissociated salt coordinate with polar groups of PVA backbone and form a charge-transfer complex, which causes the change in the structural, morphological, thermal, and electrical properties of PVA [3]. Thus, the physical and chemical properties of PVA can be tuned by the addition of salts [16]. Many researchers attempted to enhance the ionic conductivity of SPEs by incorporating different micro- or nano-sized inorganic fillers into the matrix to reduce the crystalline phase of the host polymer [17]. The suppression of crystallization in a semi-crystalline polymer host leads to enhancement in the segmental chain motion, consequently prompting better ionic conduction [18].

Zinc oxide nanoparticles (ZnO-NPs) are wide-bandgap semiconductors that possess optoelectrical characteristics. ZnO-NPs have been dispersed into several SPE systems to improve the ionic conductivity, structure and mechanical properties of the produced nanocomposite solid polymer electrolytes (NSPEs) [19]. Zebardastan et al. [20] investigated the properties of NSPEs, which consisted of different weight percentages of ZnO nano-filler in the PVdF-HFP:PEO:EC:PC:NaI:I_2_:ZnO system. The highest value of ionic conductivity was recorded when 3 wt.% of ZnO nanofiller was added into the system. They ascribed the enhancement in ionic conductivity to the increase in the amorphous portion in the system. Recently, Selvi et al. [21] studied the effect of ZnO-NP content on the optical, electrical, mechanical, and thermal properties of pure PVA. The results of the study revealed that the mechanical and thermal properties of PVA were improved upon adding ZnO-NPs.

Based on our recent published works [22,23], the proton-conducting SPE based on PVA loaded with 30 wt.% of NH_4_NO_3_ exhibits a maximum ionic conductivity of 5.17 × 10^−5^ S/cm at ambient temperature. Although the ionic conductivity of this SPE is enhanced, it is still insufficient for practical applications. In the present work, different contents of ZnO-NPs were dispersed in the proton-conducting PVA/NH_4_NO_3_ polymer electrolyte membranes to produce NSPEs with enhanced ionic conductivity. The samples were prepared by a common casting technique, and their properties with different ZnO-NP contents were compared. The prepared samples were characterized by impedance spectroscopy, where the data were analyzed as a function of composition, frequency, and temperature.

## 2. Experimental

### 2.1. Sample Preparation

**Figure 1:**
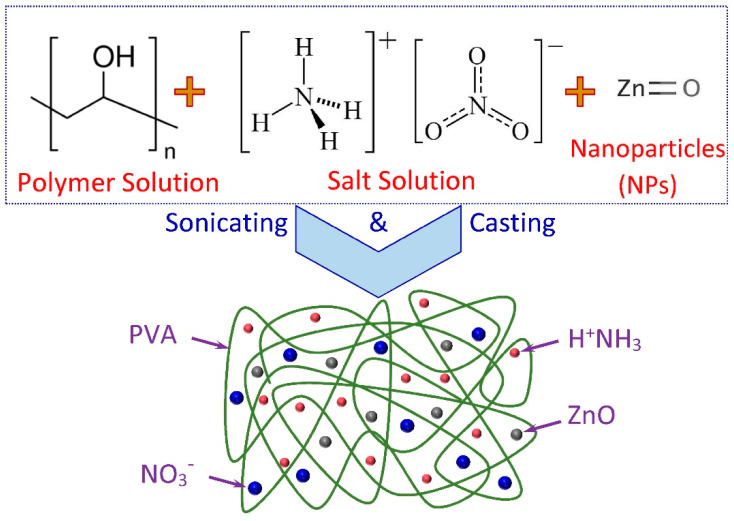
Sample preparation and characterization scheme for proton-conducting polyvinyl alcohol (PVA)/NH_4_NO_3_/ZnO nanocomposite solid polymer electrolytes (NSPEs).

Series films of proton-conducting PVA/NH_4_NO_3_/ZnO NSPEs with different ZnO-NP contents were prepared by the casting method. Referring to our previous works, the SPE based on PVA incorporated with 30 wt.% NH_4_NO_3_ exhibited the highest room-temperature ionic conductivity [22,23]. In this study, the SPE solution was prepared by dissolving 2 g of PVA in 40 mL of double-distilled water and heating for 60 min at 90 °C. The PVA solution was cool at ambient temperature, and then 30 wt.% NH_4_NO_3_ (0.857 g) dissolved separately in 5 mL was added to the viscous polymer solution, with continuous stirring for a further 45 min to ensure the intimate mixing. Later, different weight percentages of ZnO-NPs (1 to 5 wt.%) with particle sizes of 10–70 nm were added to the previous solution, and the mixtures were sonicated for 30 min to achieve homogeneous dispersion of NPs. The homogeneous solutions were then poured into various clean Petri dishes and left to dry in the air under ambient conditions for a two-week period for the membranes to form. After air-drying, the samples were stored in a desiccator with silica gel to ensure that the films were completely dried prior to subsequent experimentation and characterizations. The nanocomposite solid polymer electrolyte (NSPE) membranes were coded as NSPE-0, NSPE-1, NSPE-2, NSPE-3, NSPE-4, and NSPE-5, for a proton-conducting PVA/NH_4_NO_3_ polymer electrolyte, loaded with 0, 1, 2, 3, 4, and 5 wt.% of ZnO-NPs, respectively. The scheme for sample preparation is depicted in Figure 1.

### 2.2. Characterizations

The X-ray diffraction (XRD) pattern of the prepared proton-conducting PVA/NH_4_NO_3_/ZnO NSPEs was collected using a Bruker D8 diffractometer (Karlsruhe, Germany), with Cu-Kα radiation = 1.5418 Å, in the 2θ range between 10° and 70°. The instrument operated at 40 kV and 40 mA.

The surface morphology of the prepared samples was conducted by scanning electron microscopy (SEM, JEOL JSM-6060) (Tokyo, Japan), operating at 20 kV. The samples were sputtered with thin gold layers prior to imaging.

Electrochemical impedance spectroscopy (EIS) measurements were performed in the frequency range from 100 Hz to 2 MHz and in the temperature range 303 K to 353 K to examine the effect of ZnO-NPs on the ionic conductivity of proton-conducting PVA/NH_4_NO_3_ polymer electrolytes. The impedance was measured with a KEYSIGHT E4980A LCR Meter (Santa Rosa, CA, USA) that has been interfaced with a computer. The proton-conducting NSPE samples were mounted on the holder with aluminum blocking electrodes of diameter 1 cm under spring pressure to ensure good contact between NSPE films and electrodes. In this study, the semicircular arc of the Cole–Cole plot of complex impedance was used to obtain the bulk resistance ($R_{b}$), and the electrical conductivity (σ) of the samples was calculated from this equation:

(1) $$\sigma =\frac{l}{R_{b}A}$$

Here, l and A are, respectively, the sample thickness and the area of the electrode. A micrometer gauge was used to determine the thickness of films and was found to range from 240 to 315 µm.

## 3. Results and Discussion

### 3.1. XRD Analysis

**Figure 2:**
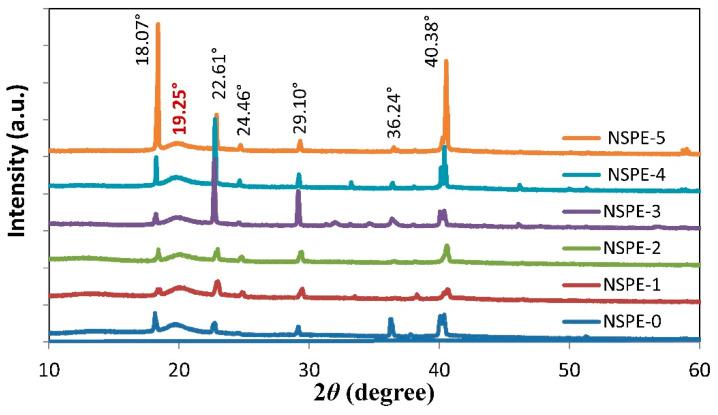
XRD pattern plots for PVA/NH_4_NO_3_/ZnO NSPEs with different ZnO-nanoparticle (NP) contents.

The XRD patterns for PVA/NH_4_NO_3_/ZnO NSPEs with varying ZnO-NP contents are shown in Figure 2. The XRD patterns for all samples exhibit a broad peak centered at 2*θ* = 19.25°, corresponding to the semi-crystalline nature of PVA that arises from the intra- and inter-molecular hydrogen bonding of the O–H groups in the PVA backbone [24]. However, the presence of NH_4_NO_3_ crystalline peaks in all samples at 18.07°, 22.61°, 24.46°, 29.10°, 36.24°, and 40.38° indicates the presence of some undissociated NH_4_NO_3_ salt in the NSPE samples [25].

The minimum intensity of the characteristic peaks upon loading 2 wt.% ZnO-NP reveals the lowest relative crystallinity of the NSPE-2 sample. Beyond this concentration, the intensity of the NH_4_NO_3_ peaks increases as the ZnO-NP concentration increases, which causes a decrement in the conductivity due to the recombination of the dissociated ions to form NH_4_NO_3_ salt. It has been well reported that even a small change in the crystallinity of polymer samples has a profound effect on the conductivity [26,27,28].

### 3.2. SEM Study

**Figure 3:**
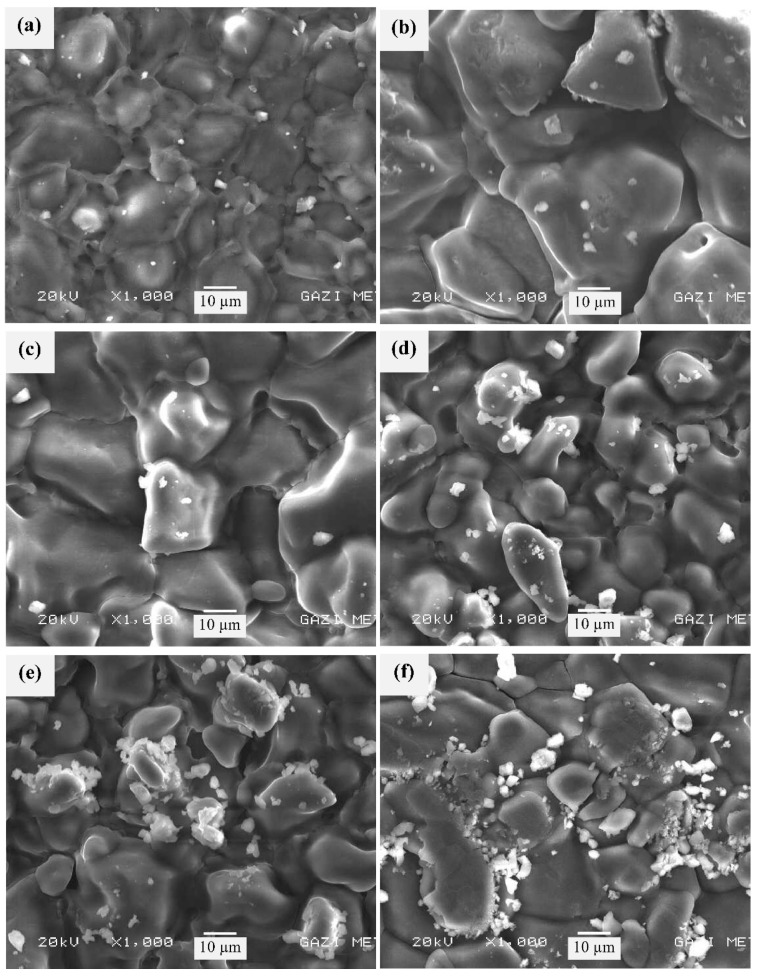
Scanning electron microscope (SEM) micrographs of PVA/NH_4_NO_3_/ZnO NSPEs with different ZnO-NP concentrations: (**a**) NSPE-0, (**b**) NSPE-1, (**c**) NSPE-2, (**d**) NSPE-3, (**e**) NSPE-4, and (**f**) NSPE-5.

Figure 3 depicts the scanning electron microscope (SEM) micrographs of PVA/NH_4_NO_3_/ZnO NSPEs with different ZnO-NP contents. It can be seen that the morphology of undoped and doped proton-conducting polymer electrolytes consists of solid structures that have protruded out of the membrane surface, revealing that the NH_4_NO_3_ salt has recrystallized out of the NSPE surface, which is in good agreement with the XRD results. It is also clear that the density of the solid structures reduced after the addition of 2 wt.% of ZnO-NPs. A further increase in ZnO-NP content resulted in a further increase in the size and density of these solid structures in the polymer electrolytes. This observation is in agreement with the aforementioned XRD analysis.

### 3.3. Impedance Analysis

**Figure 4:**
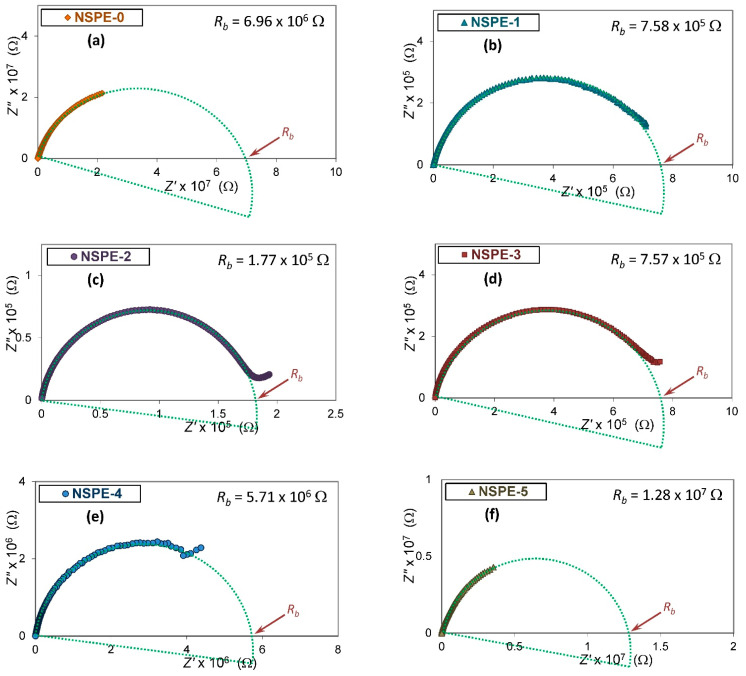
Impedance plots of PVA/NH_4_NO_3_ polymer electrolyte samples containing 0 wt.% (**a**); 1 wt.% (**b**); 2 wt.% (**c**); 3 wt.% (**d**); 4 wt.% (**e**); and 5 wt.% (**f**) of ZnO-NPs at room temperature.

Figure 4 represents the Cole–Cole plots of complex impedance for samples with 0, 1, 2, 3, 4, and 5 wt.% ZnO-NPs loaded PVA/NH_4_NO_3_ polymer electrolytes at room temperature. The profile plots show a part of a depressed semicircle for all samples. The semicircle arc symbolizes the parallel combination of bulk resistance (due to mobile ions inside the polymer matrix) and bulk capacitance (due to immobile polymer chains) [29]. As the ZnO content increases, the semicircle in the plots was observed to lessen up to 2 wt.%; beyond this concentration, the trend is reversed.

The $R_{b}$ for NSPE samples has been found from the intercept of the semicircle arc at low-frequency on the real $Z^{\prime }$ axis. As shown in the inset of Figure 4, the $R_{b}$ has been found to be 6.92 × 10^6^, 7.36 × 10^5^, 1.79 × 10^5^, 7.51 × 10^5^, 5.64 × 10^6^, and 1.15 × 10^7^ Ω for 0, 1, 2, 3, 4, and 5 wt.% content of ZnO-NPs, respectively.

The conductivity of proton-conducting PVA/NH_4_NO_3_/ZnO NSPE membranes was observed to increase with temperature. At high temperatures, the dissociation of ammonium nitrate and the thermal movement of PVA molecular chain segments would be improved, which caused an increase in the ionic conductivity.

**Figure 5:**
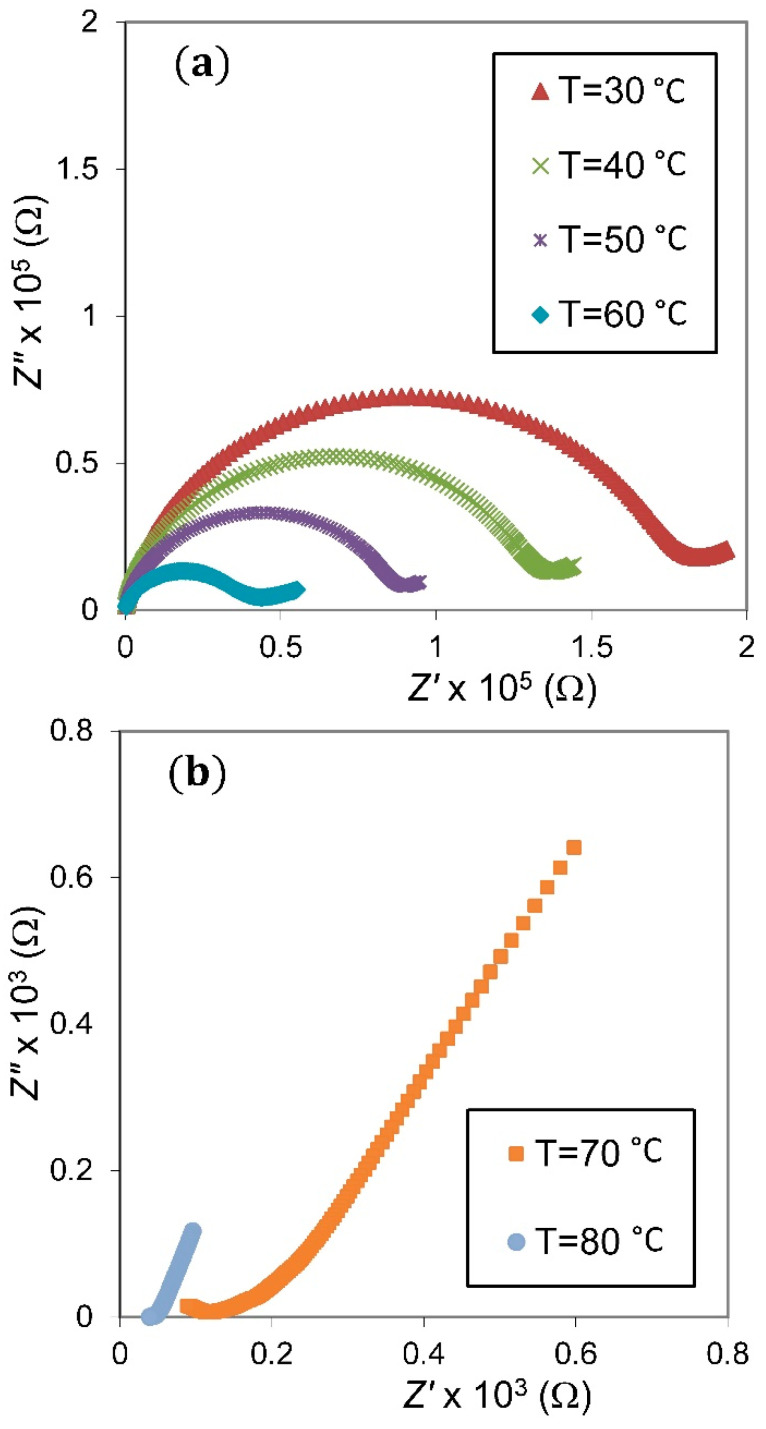
Impedance plot of the NSPE-2 sample at temperatures of 30–60 °C (**a**) and 70–80 °C (**b**).

The complex-plane impedance plots of the NSPE-2 sample at different temperatures are presented in Figure 5. It is obvious that, as the temperature increased, the depressed semicircular arc in the plots was observed to lessen and finally disappear, leaving only a low-frequency spike. This suggests the existence of only the resistive component [29,30], which reveals the absence of capacitive nature; therefore, only the diffusion processes take place at high temperatures [31].

### 3.4. Conductivity Analysis

**Figure 6:**
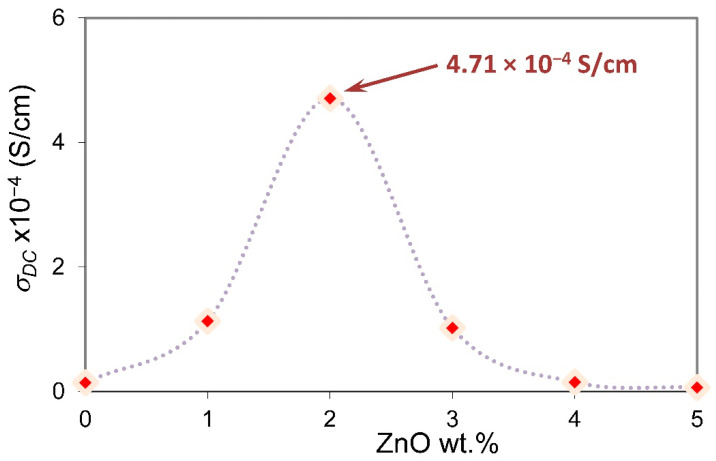
Ionic conductivity as a function of ZnO wt.% at room temperature.

The variation in room-temperature direct current (DC) conductivity ($\sigma_{DC}$) versus ZnO-NP content is presented in Figure 6. The dependence of $\sigma_{DC}$ on ZnO content provides information on the particular interaction between ions of NH_4_NO_3_ salt and the functional group of the PVA matrix. Kadir et al. [32] reported that the chitosan-PVA–NH_4_NO_3_ proton-conducting system gives the optimum value of ionic conductivity 2.07 × 10^−5^ S/cm at room temperature upon incorporating 40 wt.% salt, which is comparable with the undoped SPE sample in the present work. The peak observed in Figure 6 depicts the room-temperature highest DC conductivity optimized at 4.71 × 10^−4^ S/cm with the addition of 2 wt.% of ZnO-NPs. Beyond that, the ionic conductivity decreases quickly. Tripathi and Kumar [33] attributed the decrease in conductivity with increasing ZnO concentration beyond 3 wt.% in plasticized polymer gel electrolytes based on poly(vinylidene fluoride)-co-hexafluoropropylene (PVDF-HFP) to the small value of ZnO-NPs′ dielectric constant compared to the polymer gel electrolyte system.

The average value of $\sigma_{DC}$ for all prepared samples is tabulated in Table 1. The increases in $\sigma_{DC}$ with the addition of ZnO-NPs could be related to the increase in both the number and mobility of free carriers in the matrix by increasing the degree of salt dissociation of ion aggregates, and increasing the amorphous phase content, respectively [34,35].

**Figure 7:**
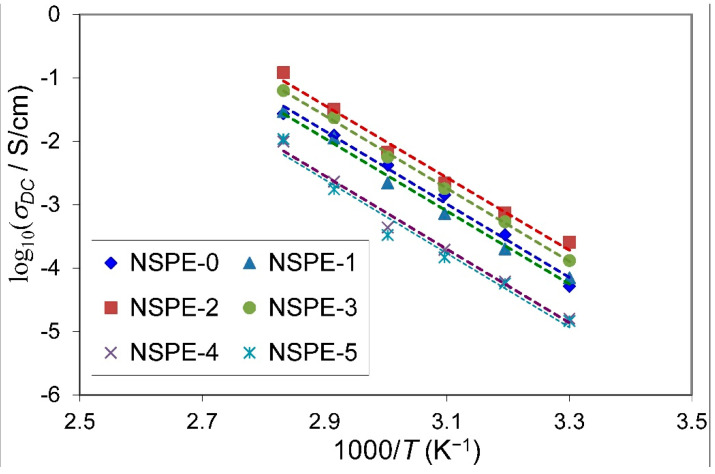
Temperature-dependent direct current (DC) conductivity for PVA/NH_4_NO_3_/ZnO NSPEs.

The temperature-dependence of ionic conductivity has been employed to analyze the possible ion-conduction mechanism in the present proton-conducting NSPEs. Figure 7 shows the plot of $\log\sigma_{DC}$ versus 1000/T for different ZnO-NP contents in PVA/NH_4_NO_3_ polymer electrolyte membranes. The linear variation of these plots suggests that the thermally activated process exhibits the Arrhenius-type behavior [36]. However, the observed linear relations for all doped PVA/NH_4_NO_3_ polymer electrolyte samples mean that there is no phase transition marked in the NSPEs by adding ZnO-NPs. As per this model, the temperature-dependent $\sigma_{DC}$ can be expressed by activation energy ($E_{A}$), which is obtained in terms of the Arrhenius equation [37]:

(2) $$\sigma_{DC}=\sigma_{o}\exp\left(\frac{-E_{A}}{k_{B}T}\right)$$

where $\sigma_{o}$ and $k_{B}$ represent the pre-exponential factor and Boltzmann constant, respectively. The value of $E_{A}$ was calculated using the grade of the Arrhenius plot shown in Figure 7.

The increase in the $\sigma_{DC}$ of NSPEs with temperature can be understood as the hopping of proton ions between PVA coordinating sites; hopping being helped by both polymer chain segmental motions and local structural relaxations [38]. As the temperature increases, the amorphous domains gradually increase, and the polymer chains earn faster internal modes producing segmental motion due to bond rotations. As a result, ion hopping due to inter- and intra-chain movements is favored, causing the spectacular to enhance the conductivity of the matrix [39,40]. The calculated values of $\sigma_{DC}$ and $E_{A}$ in accordance with ZnO content are presented in Table 1, which also shows that the $E_{A}$ is inversely proportional to the $\sigma_{DC}$ in the manner that the highest conductivity sample (NSPE-2) shows the minimum value of hopping activation energy. Here, the $E_{A}$ has been assigned as the energy acquired by the H^+^ ion to free itself from its localized-state. This reveals that incorporating a small amount (2 wt.%) of ZnO-NPs into the PVA/NH_4_NO_3_ polymer electrolyte causes a reduction in the potential energy barriers for the proton transport, leading to a decrease in the activation energy [41]. This result is expected and comparable with much previous work for different proton-conducting polymer electrolyte systems [29,34,42]. Hema et al. [43] also observed that the temperature-dependent conductivity for the proton-conducting polymer electrolyte based on PVA–NH_4_Cl, PVA–NH_4_Br, and PVA-NH_4_I followed the Arrhenius-type relationship.

### 3.5. Dielectric Study

The dielectric study of the present proton-conducting NSPEs was carried out to understand the conductivity behavior of the systems and is explained in terms of the real ($M^{\prime }$) and imaginary ($M^{\prime \prime }$) parts of electric modulus, as they are free from the contribution of the interfacial electrode/electrolyte polarization effect at low frequencies. The dielectric study gives information on relaxing dipoles in the samples [44]. The obtained complex permittivity ($\varepsilon^{*}$) data were analyzed using complex modulus ($M^{*}$), which is an inverse of the $\varepsilon^{*}$ and is linked to the impedance data as follows:

(3) $$M^{*}=\frac{1}{\varepsilon^{*}}=j\omega C_{o}Z^{*}$$

Here, ω is the angular frequency (ω=2πf, f being frequency), $C_{o}=\varepsilon_{o}A/d$, where $\varepsilon_{o}$ is the free space permittivity, A is the cross-section of the electrode, and d is the film thickness, and $Z^{*}$ is the complex impedance.

**Figure 8:**
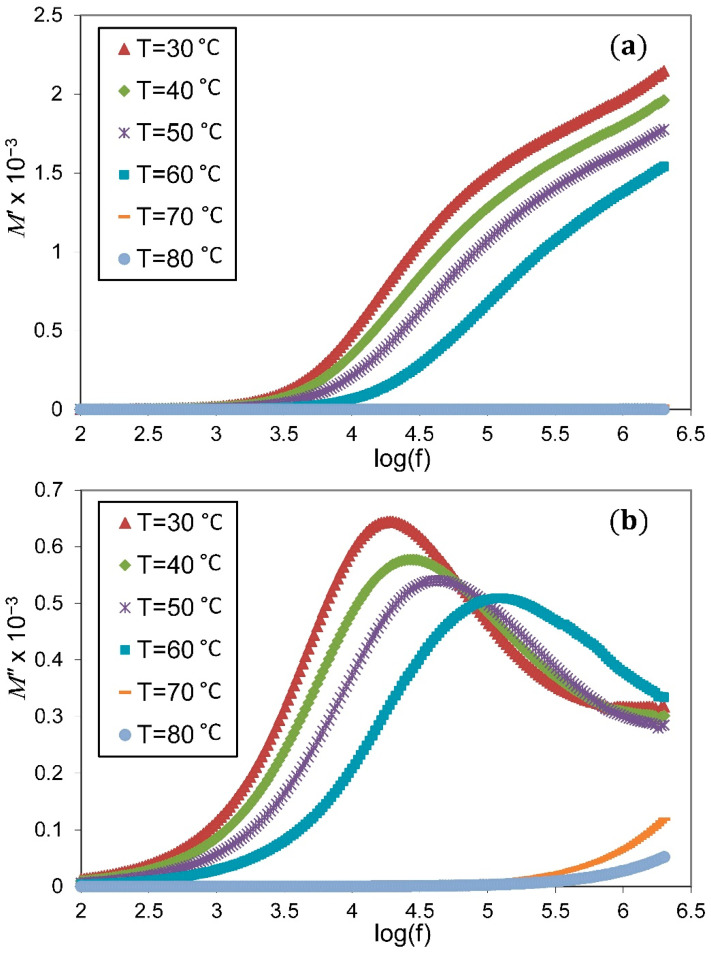
Plot of (**a**) real ($M^{\prime }$) and (**b**) imaginary ($M^{\prime \prime }$) parts of electric modulus versus frequency for the PVA/NH_4_NO_3_ doped 2 wt.% ZnO at various temperatures.

The variations of real ($M^{\prime }$) and imaginary ($M^{\prime \prime }$) parts of electric modulus for the PVA/NH_4_NO_3_ doped 2 wt.% ZnO at various temperatures are introduced in Figure 8. The plot of $M^{\prime }$ and $M^{\prime \prime }$ shows low value at lower frequencies, which is caused by the huge value of interfacial capacitance correlated with the electrode-electrolyte boundary [34]. However, no definitive peaks can be observed for the $M^{\prime }$ plot, and the $M^{\prime \prime }$ spectrum shows an asymmetry relaxation peak accompanied by the dispersion of $M^{\prime }$ in the frequency range employed in this study. The broadness and asymmetry shape of the $M^{\prime \prime }$ peak disclose the distribution of relaxation time and non-Debye relaxation process [14,45].

A shift in the $M^{\prime \prime }$ relaxation peak towards the higher-frequency side with a temperature rise indicates the reduction in the relaxation time, which directly supports the ionic conductivity enhancement as a consequence of an increase in the mobility of free ions [17]. According to Khiar and Arof [46], as temperature increases, the degree of salt dissociation and re-dissociation of ion aggregates causes an increase in the number of free ions.

**Figure 9:**
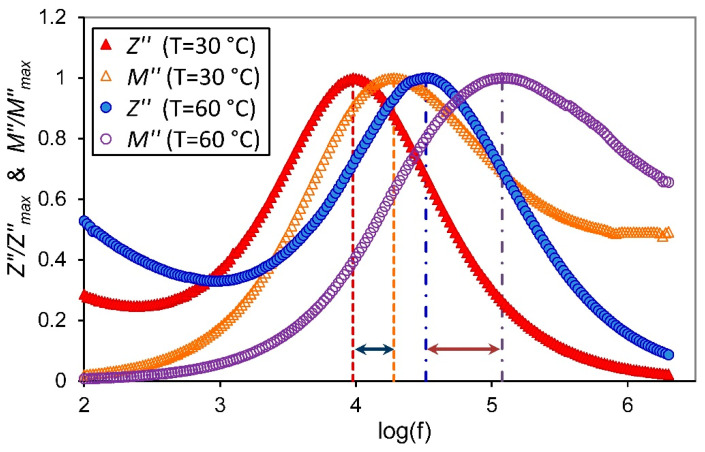
The change of normalized imaginary parts of impedance ($Z^{\prime \prime }/Z^{\prime \prime }{}_{max}$) and electric modulus ($M^{\prime \prime }/M^{\prime \prime }{}_{\max}$) versus frequency at temperatures of 30 °C and 60 °C for the PVA/NH_4_NO_3_ doped 2 wt.% ZnO-NPs.

The combined plots of $Z^{\prime \prime }$ and $M^{\prime \prime }$ against frequency are usually used to identify whether the short-range or long-range motion of free carriers is dominant in the relaxation process. The mismatch of frequency peaks between $Z^{\prime \prime }$ and $M^{\prime \prime }$ reflects that the short-range movement of free carriers is the predominant process and departs from the ideal Debye-type model, whereas the coincidence of the frequency peaks at a similar frequency implies that the long-range movement of free carriers is dominant [47]. The frequency response of normalized $Z^{\prime \prime }/Z^{\prime \prime }{}_{\max}$ and $M^{\prime \prime }/M^{\prime \prime }{}_{\max}$ for the sample with 2 wt.% of ZnO-NPs at temperatures 30 and 60 °C was represented in Figure 9. From this figure, it is noticed that the $Z^{\prime \prime }/Z^{\prime \prime }{}_{\max}$ and $M^{\prime \prime }/M^{\prime \prime }{}_{\max}$ peaks do not concur, indicating the short-range movement of free carriers and non-Debye relaxation processes in the present proton-conducting NSPE sample. The mismatch between the $Z^{\prime \prime }/Z^{\prime \prime }{}_{\max}$ and $M^{\prime \prime }/M^{\prime \prime }{}_{\max}$ peaks become larger with increasing temperature, which suggests the increases in the portion of the short-range movement of free carriers with increasing temperature. This result is in accordance with previous works, which suggest that the conductivity increases with increasing temperature.

### 3.6. Ion Transport Parameters

Ion transport parameters such as charge carrier density (n), its mobility (μ), and the diffusion coefficient (D) of the present proton-conducting PVA/NH_4_NO_3_/ZnO NSPE membranes are investigated in detail using the Rice–Roth model [48]. This model postulated that the ionic carrier of mass m in the localized states could be thermally excited to a free-ion-like state after receiving energy equal to the activation energy of conduction ($E_{A}$); wherein the ion is propagated through the electrolyte with a velocity (v) [49], given by: $v=\sqrt{2E_{A}/m}$. The mean free path of ion transport or the distance traveled by the ion between two complexation sites (ℓ) is given as: ℓ=vτ, where τ is the time of ions′ travel from one complex site to another. In the present work, ℓ is the hopping distance between two repeating units of hydroxyl groups in PVA, which is taken to be around 2.15 Å [50,51].

According to the Rice–Roth model, the ionic conductivity of free mobile ion is expressed as:

(4) $$\sigma =\frac{1}{3}\left[\frac{{\left(Ze\right)}^{2}}{k_{B}T}\right]n\ell v\exp\left(-\frac{E_{A}}{k_{B}T}\right)$$

Here, Z is the valency of the conducting ions, e is the electron charge, and $k_{B}$ is the Boltzmann constant. Equation (4) was used to evaluate the number density of mobile ions (n). From the estimated value of n, the ionic mobility (μ=σ/ne) and diffusion coefficient ($D=k_{B}T\sigma /ne^{2}$) of the samples can also be calculated. Table 1 shows the value of n, μ, and D for the PVA/NH_4_NO_3_/ZnO NSPEs samples with different ZnO contents. It is seen from the table that the maximum conducting sample has a maximum value of n and μ, which confirms that the conductivity in the present NSPEs is actually controlled by both the number and mobility of H^+^ ions in the samples. These studies indicate that the conductivity of the PVA/NH_4_NO_3_ polymer electrolyte can be enhanced moderately by adding a small percentage of ZnO-NPs, owing to the increase in both the mobility and number density of mobile proton ions.

## 4. Conclusions

Proton-conducting PVA/NH_4_NO_3_/ZnO NSPE membranes with different contents of ZnO-NPs were prepared using the cast technique. The small percentage of ZnO-NPs was found to influence the proton-conduction of the system, and the highest obtained value of conductivity is 4.71 × 10^−4^ S/cm at room temperature. The temperature-dependent ionic conductivity results exhibited Arrhenius behavior, and the activation energy values were inversely proportional to the DC conductivity. Dielectric studies suggest that the NSPE samples in this study exhibit non-Debye behavior, and the relaxation process is caused by the short-range movement of free carriers. The application of the Rice–Roth model deduced that the increase in conductivity arose from the increase in the mobility and number density of mobile proton ions in the system.

## Figures and Tables

**Table 1:** Transport parameters for PVA/NH_4_NO_3_/ZnO nanocomposite solid polymer electrolytes (NSPEs) samples at room temperature.

Samples	$\sigma_{DC}$ × 10^−4^ (S cm^−1^)	$E_{A}$ (eV)	τ × 10^−14^ (s)	n × 10^18^ (cm^3^)	µ × 10^−4^ (cm^2^ V^−1^ s)	D × 10^−8^ (cm^2^ s^−1^)
NSPE-1	1.133	0.912	3.64	4.71	1.50	3.93
NSPE-2	4.710	0.906	3.65	18.8	1.57	4.10
NSPE-3	1.021	0.914	3.63	4.28	1.49	3.90
NSPE-4	0.152	0.920	3.62	0.67	1.42	3.71
NSPE-5	0.067	0.925	3.61	0.31	1.38	3.60

## Data Availability

The authors confirm that the data supporting the findings of this study are available within the article.
